# Concomitant visceral and localized cutaneous leishmaniasis in two Moroccan infants

**DOI:** 10.1186/s40249-018-0413-8

**Published:** 2018-04-12

**Authors:** Tarik Mouttaki, Hasnaa Maksouri, Jilali El Mabrouki, Gema Merino-Espinosa, Hassan Fellah, Mohamed Itri, Joaquina Martin-Sanchez, Maha Soussi-Abdallaoui, Soumiya Chiheb, Myriam Riyad

**Affiliations:** 1 Centre of Doctoral Studies on Health Sciences (CED. des Sciences de la Santé), Doctoral School of Immunopathology, Faculty of Medicine and Pharmacy, Hassan II University of Casablanca, Casablanca, Morocco; 2 Research Team on Immunopathology of Infectious and Systemic Diseases, Faculty of Medicine and Pharmacy, Hassan II University of Casablanca, Casablanca, Morocco; 3 Laboratory of Parasitology and Mycology, University Hospital Ibn Rochd of Casablanca, Casablanca, Morocco; 4 Department of Parasitology, Faculty of Pharmacy, University of Granada, Granada, Spain; 5 Children’s Hospital, University Hospital Ibn Rochd of Casablanca, Casablanca, Morocco; 6 Laboratory of Parasitology, Faculty of Medicine and Pharmacy, Hassan II University of Casablanca, 19 rue Tarik Ibn Ziad, BP. 9154 Casablanca, Morocco; 7 Department of Dermatology, University Hospital Ibn Rochd of Casablanca, Casablanca, Morocco

**Keywords:** Visceral leishmaniasis, Cutaneous leishmaniasis, *Leishmania infantum*, *Leishmania tropica*, Infants, Morocco

## Abstract

**Background:**

Leishmaniases are vector-borne diseases caused by the protozoa of the *Leishmania* genus. The clinical spectrum of these diseases extends from benign dermal lesions to visceral forms. In the Mediterranean region, zoonotic visceral leishmaniasis (ZVL) is caused by *L. infantum.* If untreated within two years, the disease usually leads to death. In Morocco, ZVL is endemic in the north, with a hundred cases notified each year, mostly in children aged below five years. Here, we report on two clinical observations in infants presenting unusual concomitant VL and cutaneous leishmaniasis (CL) in Morocco.

**Case presentation:**

In this case study, we report on two infants aged nine and 12 months old. They both have a history of febrile splenomegaly, anemia, and pallor of mucous membranes. Visceral leishmaniasis was confirmed by parasitological diagnosis (positive bone marrow smear and screening of anti-*L. infantum* antibodies). However, the clinical examination also showed cutaneous lesions that suggested the presence of CL. This was reinforced by the patients having a history of living or traveling to endemic foci. Thus, direct examination, culture, and PCR-RFLP (ITS1-Hae 3) were carried out on the patients’ dermal exudates. In one of the infants, CL was associated with *L. infantum*, while in the other it was associated with *L. tropica*. The infants were treated as according to the recommendations of the Ministry of Health. Both patients were cured in two months; defervescence, reduction of splenomegaly, and healing of cutaneous lesions were all observed.

**Conclusions:**

These singular patients illustrate the clinical polymorphism of CL and the necessity of updating the differential diagnosis of leukemia-like syndromes, including VL, in children living in or travelling to known endemic areas. These observations suggest a change in the Mediterranean VL phenotype that may be associated with CL.

**Electronic supplementary material:**

The online version of this article (10.1186/s40249-018-0413-8) contains supplementary material, which is available to authorized users.

## Multilingual abstracts

Please see Additional file 1 for translation of the abstract into the five official working languages of the United Nations.

## Background

Leishmaniases are vector-borne parasitic diseases caused by the flagellated protozoa of the *Leishmania* genus. They are prevalent in 98 countries and three territories on five continents [1]. The spectrum of these diseases extends from benign cutaneous lesions that heal spontaneously to visceral forms [2]. Visceral leishmaniasis (VL) is a fatal systemic disease, if left untreated. It is caused by the *Leishmania* (*L.*) *donovani* complex in East Africa and the Indian subcontinent and by *L. infantum* in Europe, North Africa, and Latin America [3].

Zoonotic VL due to *L. infantum* is endemic in the Mediterranean region [4]. If untreated within two years, the disease usually leads to death, with approximately 300 000 new VL cases occurring each year globally [1]. In Morocco, the first case of VL was described in 1922 and in 2014, 86 VL cases were notified to the Ministry of Health [5, 6]. The disease is endemic in the north of the country, with a hundred cases reported per year, mostly in children [6–8]. The disease is more threatening in children than in adults due to the relative immaturity of a child’s immune system, and is lethal if untreated [9].

In Morocco, cutaneous lesions due to *Leishmania* have never been reported in a patient with zoonotic VL. In contrast, 10–20% of patients develop cutaneous lesions (Post Kala azar Dermal Leishmaniasis) as a sequel of kala azar due to *L. donovani* [7, 10].

In this case study, we report on two retrospective pediatric observations of unusual clinical presentations with concurrent VL and cutaneous leishmaniasis (CL).

## Case presentation

This retrospective analysis concerned two pediatric patients diagnosed in 2012. Demographic data (age, sex, place of residence, occupation for adults), diagnosis, and clinical parameters were collected through a standardized information sheet.

### Patient 1

A nine-month-old girl was admitted to the Children’s Hospital (University Hospital Ibn Rochd of Casablanca) with a seven-month history of febrile hepatosplenomegaly (39 °C), anemia, pallor of mucous membranes, and pancytopenia. She was living in Casablanca but her parents reported a one-month stay in a northern endemic area of VL the previous summer.

The parasitological diagnosis (microscopic examination of bone marrow aspirate) showed the presence of *Leishmania* amastigotes. The screening for antibodies against *L. infantum* (*Leishmania* IFA IgG, Vircell, Granada, Spain) was also positive (title: 1/320; cut-off value: 1/40) (see Table 1).

**Table 1:** Results of the biological examinations carried out on patients 1 and 2

Biological examinations	Patient 1	Patient 2
VL diagnosis		
- Bone marrow stained smear	+	+
- Anti-*L. infantum* antibodies (IFAT)^a^	+ (1/320)	+ (1/80)
- Anti-*L. infantum* antibodies (WB)	ND	+ (14–16 kD)
CL diagnosis		
- Skin stained smear	–	+
- Culture (NNN)	–	+
- PCR-RFLP	*L. infantum*	*L. tropica*

+: Positive; −: Negative; ND: Not done

^a^Cut-off value: 1/40

Furthermore, the clinical examination showed two cutaneous lesions of 5 mm diameter: one evolutive erythematous papule on the face and one scar lesion on the arm. The aspect of these lesions and the history of travel in a known endemic area of CL due to *L. infantum* or *L. tropica* suggested CL lesions. To confirm this hypothesis, dermic exudate was sampled from the active lesion. The microscopic examination of the corresponding stained smear was negative (absence of *Leishmania* amastigotes), as was the culture on Novy – Mc Neal – Nicolle (NNN) medium (absence of *Leishmania* promastigotes after one month). The genotyping by polymerase chain reaction-restriction fragment length polymorphism (PCR-RFLP) (ITS1 – Hae 3) carried out directly on the dermal exudate according to Schönian et al. (2003) and Mouttaki et al. (2014) protocols showed *L. infantum* [11, 12] (see Table 1).

Meglumine antimoniate (Glucantime®) was administered intramuscularly at the recommended dosage by the Ministry of Health, i.e. 20 mg of pentavalent antimony (Sb5+) per kg per day for 20 days [7]. The patient was cured in two months; defervescence, reduction of the spleen size, hematological restoration, negativation of the parasitological control on bone marrow, and healing of cutaneous lesions were all observed.

### Patient 2

A one-year-old girl was admitted to the Children’s Hospital (University Hospital Ibn Rochd of Casablanca) with an impaired general condition and a three-month history of febrile (39 °C) hepatosplenomegaly, anemia, and pallor of mucous membranes. She was from an endemic area of both VL due to *L. infantum* and CL due to *L. tropica* (center of Morocco).

The bone marrow sample tested positive for *Leishmania* amastigotes. Screening utilizing the indirect fluorescent antibody technique (IFAT) for antibodies against *L. infantum* (*Leishmania* IFA IgG, Vircell, Granada, Spain) was weakly positive (title: 1/80; cut-off value: 1/40). This latter low value and the altered general status led us to carry out a specific western blot (WB) screening for anti-*L. infantum* antibodies (LEISHMANIA Western Blot Ig G, LDBIO Diagnostics, Lyon, France) and a screening for anti-HIV antibodies. Only the *L. infantum* serology by WB was positive (antibodies anti-14–16 kD proteins) (see Table 1).

Moreover, the clinical examination revealed two cutaneous papule lesions evolving for three months on the face, which did not heal with a previously prescribed dermal antibiotherapy before the patient’s hospital admission. These lesions as well as the infant’s origin from a known endemic area of CL due to *L. infantum* and/or *L. tropica* led us to sample the dermal exudate. The corresponding stained smear was positive as was the culture on NNN medium. The genotyping by PCR-RFLP carried out on the dermal sample and the isolate revealed *L. tropica*.

Meglumine antimoniate (Glucantime®) was administered following the same protocol as for patient 1, but for 30 days. The patient was cured after two months of treatment; defervescence, reduction of splenomegaly, and healing of cutaneous lesions were all observed.

## Discussion and conclusions

In South Europe and North Africa, the Mediterranean type of VL is usually caused by *L. infantum*, and infants or children aged up to four years are frequently affected. The incubation period ranges from 10 days to over one year, and the disease onset is usually gradual. The common symptoms are fever, malaise, shivering or chills, weight loss, anorexia, and discomfort in the left hypochondrium. The common clinical signs are non-tender splenomegaly, with or without hepatomegaly, wasting and pallor of mucous membranes. Signs of malnutrition (edema, skin and hair changes) develop as the disease progresses. Intercurrent infections are common [13].

Clinically, both infants observed in this study presented clinical signs suggestive of VL. This hypothesis was reinforced by the fact that they either lived or stayed in known endemic VL foci according to Ministry of Health data. The positive bone marrow smear and the anti-*L. infantum* serology allowed us to confirm VL in both cases. These children were then treated with Glucantime® and were cured within two months. In Morocco, the recommended treatment for VL is antimonials (20 mg Sb+/kg/day for three weeks), and the cure rate is 99% [7, 14].

Furthermore, both infants presented signs suggestive of CL, as follows: cutaneous lesions on exposed skin surfaces (face and arm), history of resistance to dermal antibiotic treatment, and history of living or traveling the previous summer to known endemic foci of CL due to *L. tropica* or *L. infantum*. While the conventional parasitological diagnostic methods (dermal smears and culture) confirmed the diagnosis only for patient 2, the genotyping on the dermal samples revealed *L. infantum* in patient 1 and *L. tropica* in patient 2.

In Morocco, VL is a zoonotic rural disease endemic in northern regions and cases are sporadically reported in some southern regions. *Leishmania infantum* is also responsible for sporadic CL in North Africa in the same geographical area as VL [15]. In Morocco, the first *L. infantum* CL patient was reported in 1996 from an active focus of canine VL in the northern Rif mountains [16]. In fact, most CL cases due to *L. infantum* are notified from one focus (Sidi Kacem in the northwest), where sporadic cutaneous forms have been described as two-year-course single lesions [17–19]. To our knowledge, the association of VL and CL in the same human immunocompetent host has not been previously described.

*Leishmania tropica* is responsible for anthroponotic CL in Morocco. In the 1980s, the disease was hypoendemic in center-south rural foci. In the 1990s, urban epidemic foci appeared in the north [20, 21]. In fact, CL due to *L. tropica* has the widest geographic distribution in the country and the highest incidence in North Africa [8, 15, 22].

The CL cases presented in this study are interesting due to their association with a visceral syndrome. Until now, the clinical definition of VL in Morocco by the Ministry of Health highlighted the absence of cutaneous lesions. Thus, *L. infantum* could be responsible for both the visceral and cutaneous symptomatology in patient 1, whereas two different species, *L. infantum* and *L. tropica*, could be respectively responsible for the visceral and cutaneous symptomatology in patient 2.

For patient 1 (CL due to *L. infantum*), the symptomatology could be explained either by the inoculation of different viscerotropic and dermotropic strains of *L. infantum* through the vector bite, or by the immunological status of children, which would have allowed the visceral dissemination of a dermotropic strain of *L. infantum*. Patient 2, who had CL due to *L. tropica*, lived in a co-endemic region of VL due to *L. infantum* and CL due to *L. tropica*. We could not discriminate between *L. tropica* and *L. infantum* on the bone marrow. We therefore cannot state with certainty that this patient’s VL was due to *L. infantum*. Few cases of VL caused by *L. tropica* have been reported in India, Iran, and Israel [10, 13, 23].

Furthermore, IFAT serology showed a low antibody title (1/80) close to the cut-off value in patient 2, which is consistent with a cross-reaction. Cross-reactions have also been described with *L. tropica* using WB [24]. If the VL of this patient was caused by *L. infantum*, this would highlight the possibility of a coinfection by two endemic species in the same focus. It is usually considered that vector and human hosts are infected by a single species and that the natural infection by one *Leishmania* species would protect against reinfections by homologous or heterologous species [25, 26]. However, natural human coinfections have been reported in Bolivia, Brazil, Iran, Iraq, Mexico, and Peru [25, 27]. In Morocco, these observations must be kept in mind due to the increasing number of reported co-endemic CL foci due to *L. infantum* and *L. tropica*, or *L. tropica* and *L. major* [8, 17].

Finally, these patients illustrate the clinical polymorphism of CL and its difficult clinical diagnosis, even in endemic regions. They also highlight the importance of updating the differential diagnosis of leukemia-like syndromes, including VL in infants living in or travelling to known endemic areas. Indeed the patients staying during the summer in known foci was a pivotal anamnestic fact that directed the clinical diagnosis towards VL and then CL that were confirmed by the biological examinations. Our observations also suggest a change in the Mediterranean VL phenotype that may be associated to cutaneous lesions. It is thus important to characterize *Leishmania* species in any unusual clinical situation and to report particular cases such as coinfections in order to contribute to better epidemiological and physiopathological knowledge of leishmaniases in endemic areas.

## Additional file

Additional file 1: Multilingual abstracts in the five official working languages of the United Nations. (PDF 346 kb)

## Abbreviations

CL: Cutaneous leishmaniasis
IFAT: Indirect fluorescent antibody technique
PCR-RFLP: Polymerase chain reaction-restriction fragment length polymorphism
VL: Visceral leishmaniasis
WB: Western blot
ZVL: Zoonotic visceral leishmaniasis
