# Supplementary material for: Concomitant visceral and localized cutaneous leishmaniasis in two Moroccan infants
Source: Infect Dis Poverty. 2018 Apr 12;7:32. doi: 10.1186/s40249-018-0413-8 (PMC5896130; doi:10.1186/s40249-018-0413-8)
Supplement: Supplementary file 1 — Multilingual abstracts in the five official working languages of the United Nations. (PDF 346 kb) [file 40249_2018_413_MOESM1_ESM.pdf]

Translation of the abstract into the five official working languages of the United Nations

اقتران حالتا ليشمانيا حشوية وجلدية في طفلين مغربيين

طارق متكي، حسناء مكسوري، الجيلالي المبروكي، جيما ميرينو-إسبينوسا، حسن فلاح، محمد عتري، خواكوينا مارتن-سانشيز، مها سوسي-عبداللاوي، سمية شيهب، ميريام رياض

الملخص
خلفية: الليشمانيا من الأمراض المتناقلة بواسطة الحشرات، يسببها الحيوان الأوالي من جنس ليشمانيا وتتنوع الصورة الإكلينيكية لهذه الأمراض من آفات جلدية حميدة إلى الصورة الحشوية. في منطقة البحر المتوسط، نوع الليشمانيا الطفلية هو المسؤول عن حالات الليشمانيا الحشوي المنتقل من الحيوانات للإنسان.إذا لم يتم علاج المرض خلال سنتين، فإنه يؤدي إلى الوفاة في العادة. في المغرب، تستوطن الليشمانيا الحشوية المنقولة من الحيوانات في المناطق الشمالية، حيث يُبلغ سنويا عن مئة حالة، أغلبها من الأطفال دون خمس سنوات من العمر. نستعرض هنا ملاحظتين إكلينيكيتين متعلقة بمواليد يُعرضون للمستشفيات في المغرب مصابون بحالتي الليشمانيا الحشوية والجلدية في ذات الوقت
الصورة الإكلينيكية للحالة: في تقرير الحالة الحاضر نستعرض حالة طفلين يبلغان من العمر تسعة أشهر وإثنا عشر شهرا على التوالي عانى كلاهما في الماضي من تضخم الطحال الحمّي، وفقر الدم، وشحوب الأغشية المخاطية تم تأكيد تشخيص الليشمانيا الحشوية باختبارات الطفيليات (لطاخة نخاع العظم الموجبة، بالإضافة لوجود أجسام مضادة لطفيل الليشمانيا الطفلية) كما أظهر الفحص السريري أيضا آفات جلدية تشير لوجود عدوى الليشمانيا الجلدية وقد عزز ذلك بيان المريض في تاريخه المرضي أنه قد أقام أو زار مناطق موطونة بالليشمانيا وقد تم فحص الآفات الجلدية وزراعة النضح واختباره باختبارات تفاعل البوليمرات التسلسلي وتعدد أشكال أطوال الشُدف المقتطعة (HaeIII-ITS1) في أحد الطفلين، اقترنت الليشمانيا الجلدية بطفيل الليشمانيا الطفلية، أما في الآخر فكان مقترنا بطفيل الليشمانيا المدارية. 4- عولج الطفلين بما تنص عليه توصيات وزارة الصحة وتعافى الطفلان في خلال شهرين من الزمن، حيث ظهر عليهما اختفاء الحمى، وانخفاض تضخم الطحال، كما لوحظ التئام الآفات الجلدية.
الاستنتاجات:توضح هاتان الحالتان مدى تنوع الصورة الإكلينيكية لداء الليشمانيا الجلدي وأهمية تحديث التشخيص التفريقي للمتلازمات المشابهة للوكيميا، من ضمنها داء الليشمانيا الحشوي، في الأطفال المقيمين أو الزائرين للمناطق الموطونة. تقترح هذه الملاحظات حدوث تغير في النمط الظاهري لطفيل الليشمانيا الحشوية في منطقة البحر المتوسط والذي قد يكون مقترنا بالليشمانيا الجلدية.

Translated from English version into Arabic by Suzan Alkhodair Mahmoud Sami, through

TRANSLATORS WITHOUT BORDERS

摩洛哥两例婴儿内脏和局部皮肤利什曼病案例报道

Tarik Mouttaki, Hasnaa Maksouri, Jilali El Mabrouki, Gema Merino-Espinosa, Hassan Fellah, Mohamed Itri, Joaquina Martin-Sanchez, Maha Soussi-Abdallaoui, Soumiya Chiheb, Myriam Riyad

摘要
引言：利什曼病是一种由原生动物利什曼原虫引起的媒传疾病。这些疾病的临床表现包括良性皮肤病变到内脏型。在地中海地区，人畜共患内脏利什曼病(ZVL)是由 *L. infantum* 感染引起。如果这种疾病两年内未得到治疗，通常会引发死亡。在摩洛哥，ZVL 流行于北方地区，每年有 100 个病例，患者大部分是 5 岁以下的儿童。我们报道了摩洛哥两名婴儿的临床症

状，他们患有不同寻常的伴随性利什曼病和皮肤利什曼病(CL)。
病例报告：在本案例中，我们报道了两例分别为 9 月和 12 个月大的患儿。他们的病史均包括发热性脾肿大、贫血和粘膜发白。经寄生虫学确诊为内脏利什曼病(阳性骨髓涂片和抗 *L. infantum* 抗体筛选)。然而，临床检查也显示皮肤病变，提示存在 CL。由于患儿有流行区生活或旅行史而进一步加剧病情。因此，对患者皮肤分泌物进行直接检查、培养和 PCR-RFLP (1- haeiii)检测。其中一个患儿的 CL 与 *L. infantum* 有关，而的 CL 与 *L. tropica* 有关。根据卫生部的建议进行治疗后，两名患儿均在两个月内痊愈，退热，脾肿大减少，皮肤损伤愈合。
结论：这两例罕见婴儿患者病例说明了 CL 呈临床多态性，同时进一步强调了对生活在或前往已知的流行地区的儿童的白血病样综合征(包括 VL)的鉴别诊断的必要性。这些临床观察结果表明地中海 VL 的表型变化可能与 CL 有关。

Translated from English version into Chinese by Xin-Yu Feng, edited by Jin Chen

Leishmaniose cutanée et viscérale concomitante chez deux nourrissons marocains

Tarik Mouttaki, Hasnaa Maksouri, Jilali El Mabrouki, Gema Merino-Espinosa, Hassan Fellah, Mohamed Itri, Joaquina Martin-Sanchez[4], Maha Soussi-Abdallaoui, Soumiya Chiheb, Myriam Riyad

Résumé

Contexte : Les leishmanioses sont des maladies à transmission vectorielle causées par les protozoaires du genre *Leishmania*. Leur spectre clinique comprend différentes formes allant de lésions cutanées bénignes à des atteintes viscérales. Dans la région méditerranéenne, la leishmaniose viscérale zoonotique (LVZ) est due à *L. infantum*. Cette maladie entraîne généralement la mort si elle n’est pas traitée dans un délai de deux ans. Au Maroc, elle est endémique dans le nord, où une centaine de cas sont signalés chaque année, principalement chez des enfants âgés de moins de cinq ans. Cette étude porte sur deux cas cliniques inhabituels, observés au Maroc chez des nourrissons atteints à la fois de leishmaniose viscérale et cutanée.
Présentation de cas : Le cas de deux nourrissons, âgés de 9 et 12 mois, est présenté dans cette étude. Tous deux présentaient une splénomégalie fébrile, une anémie et une pâleur des muqueuses. La leishmaniose viscérale a été confirmée par le diagnostic parasitologique (frottis de moelle osseuse positif et dépistage positif des anticorps anti-*L. infantum*). Cependant, l’examen clinique a également révélé des lésions cutanées suggérant la présence d’une leishmaniose cutanée. Ce soupçon a été étayé par le fait que les patients avaient vécu ou voyagé dans des régions d’endémie. Un examen direct, une culture et une PCR-RFLP (ITS1-Hae III) ont donc été réalisés sur les exsudats cutanés des patients. La leishmaniose cutanée était associée à *L. infantum* chez l’un des nourrissons et à *L. tropica* chez l’autre. Les deux nourrissons ont été traités suivant les recommandations du Ministère de la Santé. En deux mois, ils étaient guéris et on a observé une défervescence, la réduction de la splénomégalie et la guérison des lésions cutanées.
Conclusions : Ces cas singuliers témoignent du polymorphisme clinique de la leishmaniose cutanée et de la nécessité de mettre à jour le diagnostic différentiel des syndromes leucémoïdes, notamment

de la leishmaniose viscérale, chez les enfants qui vivent ou voyagent dans des zones d'endémie connues. Ces observations suggèrent un changement du phénotype de la leishmaniose viscérale dans le Bassin Méditerranéen, où elle pourrait être associé à la leishmaniose cutanée.

Translated from English version into French by Suzanne Assenat and Chrystelle, through

Сопутствующий висцеральный и локализованный кожный лейшманиоз у двух марокканских детей.

Тарик Муттаки, Хаснаа Максури, Хилали Эль Мабруки, Джема Мерино-Эспиноза, Хассан Феллах, Мохамед Итри, Хоакина Мартин-Санчес[4], Маха Сусси-Абдаллауи, Сумия Чихеб, Мириам Рияд

Аннотация

Краткое описание. Лейшманиозы относятся к трансмиссивным заболеваниям, вызванным простейшими рода *Leishmania.* Клинический спектр данных заболеваний включает многие виды от доброкачественных поражений кожи до висцеральных форм. В Средиземноморском регионе зоонозный висцеральный лейшманиоз (ZVL) вызывается паразитом *L. infantum.* При отсутствии лечения в течение двух лет заболевание, как правило, приводит к смерти. Зоонозный висцеральный лейшманиоз эндемичен на севере Марокко, где регистрируются около ста случаев в год, в основном у детей младше пяти лет. В данной работе мы представляем отчёт о двух клинических наблюдениях детей, демонстрирующих необычное сочетание висцерального VL и кожного лейшманиоза (CL) в Марокко.

Представление клинических случаев. В настоящем исследовании мы докладываем о двух младенцах в возрасте девяти и 12 месяцев. У обоих имеется анамнез спленомегалии с лихорадкой, анемия и бледность слизистых оболочек. Подтверждение висцерального лейшманиоза было произведено при помощи паразитологической диагностики (положительные результаты исследования мазка костного мозга и обследование на антитела к *L. infantum*). Однако, клинический осмотр также выявил поражения кожи, что предполагает наличие кожного лейшманиоза. Это было подкреплено тем, что у пациентов имелась история проживания либо посещения эндемичных очагов. Таким образом, по кожным экссудатам пациентов были проведены прямой осмотр, культура и анализ ПЦР-ПДРФ (ITS1-HaeIII). У одного из младенцев кожный лейшманиоз был вызван L. *infantum*, тогда как у другого ребёнка он был обусловлен *L. tropica*. Младенцы прошли медицинское лечение в соответствии с рекомендациями Министерства здравоохранения. Оба пациента выздоровели за два месяца; при этом наблюдалось снижение температуры, уменьшение спленомегалии и заживление кожных повреждений.

Выводы. Данные единичные заболевания демонстрируют клинический полиморфизм кожного лейшманиоза и свидетельствуют о необходимости обновления дифференциальной

диагностики симптомов, схожих с признаками лейкемии, в том числе висцерального лейшманиоза, у детей, проживающих или посещающих известные эндемичные районы. В результате настоящих наблюдений появилось предположение о возможных изменениях средиземноморского фенотипа висцерального лейшманиоза, который может быть связан с кожным лейшманиозом.

Translated from English version into Russian by Natalia Potashnik and Liudmila Tomanek, through

TRANSLATORS
WITHOUT BORDERS

Leishmaniasis cutánea localizada y visceral concomitante en dos niños marroquíes

Tarik Mouttaki, Hasnaa Maksouri, Jilali El Mabrouki, Gema Merino-Espinosa, Hassan Fellah, Mohamed Itri, Joaquina Martin-Sanchez[4], Maha Soussi-Abdallaoui, Soumiya Chiheb, Myriam Riyad

Resumen

Antecedentes: Las leishmaniasis son enfermedades transmitidas por vectores causados por protozoos del género *Leishmania* . El espectro clínico de estas enfermedades abarca desde lesiones dérmicas benignas hasta formas viscerales. En la región mediterránea, la leishmaniasis visceral zoonótica (LVZ) es provocada por el *L. infantum.* Si la enfermedad no se trata dentro de los dos años, por lo general conduce a la muerte. En Marruecos, la LVZ es endémica en el Norte, donde se notificaron un centenar de casos cada año, en su mayoría de niños menores de cinco años. Aquí presentamos dos observaciones clínicas en niños que contrajeron LV concomitante inusual y leshmaniasis cutánea (LC) en Marruecos.
Presentación del caso: En este estudio de caso, informamos acerca de dos niños de nueve y doce años. Ambos poseen antecedentes de esplenomegalia febril, anemia y palidez de las membranas mucosas. Se confirmó leishmaniasis visceral a través de diagnosis parasitológica (examen de médula ósea positivo y detección de anticuerpos anti*L. infantum*). Sin embargo, la evaluación clínica también reveló lesiones cutáneas que sugerían la presencia de LC; hecho reafirmado por los antecedentes de los pacientes de vivir o viajar a focos endémicos. Por lo tanto, se llevó a cabo una evaluación directa, cultivo y PCR-RFLP (ITS1-HaeIII) en los exudados dérmicos de los pacientes. En uno de los niños, la LC estaba ligada al *L. infantum*, mientras que en el otro al *L. tropica*. Se trató a los niños según las recomendaciones del Ministerio de Salud. Ambos pacientes se curaron en dos meses. Se observaron defervescencia, reducción de la esplenomegalia y cicatrización de las lesiones cutáneas.
Conclusión: Estos singulares pacientes ilustraron el polimorfismo clínico de la LC y la necesidad de actualizar el diagnóstico diferencial de síndromes similares a la leucemia, LV inclusive, en niños que viven o viajan a áreas endémicas identificadas. Estas observaciones sugieren un cambio en el fenotipo de la LV mediterránea posiblemente ligada a la LC.

Translated from English version into Spanish by Ana Leticia Santos and Constanza DN, through

TRANSLATORS
WITHOUT BORDERS
